# Sex differences in the relationship between Blood Pressure and Heart Rate variability in isolated Primary Hypertension

**DOI:** 10.12669/pjms.41.5.10431

**Published:** 2025-05

**Authors:** Guoliang Gao, Zhaoyi Chen, Guoping Yan, Xuefen Guo

**Affiliations:** 1 Guoliang Gao Department of Electrophysiology, Xuancheng People’s Hospital Affiliated Xuancheng Hospital, Wannan Medical College, Anhui Province, China; 2 Zhaoyi Chen Department of Gastroenterology, Xuancheng People’s Hospital Affiliated Xuancheng Hospital, Wannan Medical College, Anhui Province, China; 3 Guoping Yan Department of Electrophysiology, Xuancheng People’s Hospital Affiliated Xuancheng Hospital, Wannan Medical College, Anhui Province, China; 4 Xuefen Guo Department of Electrophysiology, Xuancheng People’s Hospital Affiliated Xuancheng Hospital, Wannan Medical College, Anhui Province, China

**Keywords:** Blood Pressure Variability (BPV), Heart Rate Variability (HRV), Sex Differences in Essential Hypertension, Autonomic Nervous Function, Primary Hypertension

## Abstract

**Objectives:**

Blood pressure variability (BPV) and heart rate variability (HRV) are well-established indicators of autonomic functioning. However, the regulation of cardiac autonomic homeostasis differs between the sexes remains unknown. In this study, we explored the association between BPV and HRV in patients with isolated primary hypertension, with an emphasis on sex-related differences. We aimed to facilitate clinical practice by informing sex-specific diagnostic and therapeutic approaches for managing primary hypertension through our study.

**Methods:**

A retrospective analysis was conducted on 189 individuals (85 males, 104 females) diagnosed with isolated primary hypertension at the Xuancheng People’s Hospital from December 2020 to March 2023. All participants underwent 24-hours ambulatory blood pressure and Holter electrocardiogram monitoring. Correlation analyses, including multiple linear regression and Pearson/Spearman tests, were conducted to assess the relationship between BPV and HRV, with an emphasis on sex-based comparisons.

**Results:**

Among males, the 24-hours standard deviation of systolic blood pressure (24hSSD) was significantly associated with age, plasma glucose levels, and HRV metrics, including rMSSD and total power (p < 0.05). Conversely, in females, 24hSSD was positively linked to age and negatively associated with the heart rate and pNN50, which represents the percentage of RR intervals exceeding 50 ms. The data indicated stronger BPV-HRV correlations in males than in females.

**Conclusions:**

Distinct sex-based differences were identified in the relationship between BPV and HRV in primary hypertension. Males demonstrated stronger interconnections between these variables, while the associations in females were comparatively weaker. These findings highlight the importance of developing tailored, sex-specific management strategies to enhance the treatment and outcomes for individuals with primary hypertension.

## INTRODUCTION

Primary hypertension is the leading cause of death among cardiovascular diseases, thereby imposing a significant burden on public health. The number of individuals affected by hypertension has risen from 594 million in 1975 to 1.28 billion in 2023.^1^ Globally, approximately 10.8 million people have succumbed to hypertension,^2^ with a >75% lifetime risk of developing hypertension.^3^ Effective blood pressure control is the pivotal first step in mitigating the morbidity and mortality attributed to hypertension, with its benefits extending across different races and sexes.^4^,^5^ Primary hypertension is characterized by disrupted cardiovascular homeostasis resulting from various factors, which ultimately leads to endothelial dysfunction and an altered cardiovascular autonomic balance.^6^,^7^

Both blood pressure variability (BPV) and heart rate variability (HRV) are well-established indicators of autonomic functioning.^8^ However, the regulation of cardiac autonomic homeostasis differs between the sexes. Recent research has revealed notable gender disparities in the relationship between BPV and HRV, offering valuable insights into hypertension diagnosis and treatment strategies. For instance, past studies have indicated distinct autonomic nervous system regulation patterns between sexes. For instance, males tend to exhibit a predominance of sympathetic nervous system activity, while the parasympathetic activity is more pronounced in females.^3^ This was highlighted in the *Framingham Heart Study*, which reported lifetime risks for developing hypertension and emphasized these autonomic differences.

Furthermore, a study published in *Hypertension* in April 2024 examined gender-related variations in cardiovascular outcomes among elderly individuals with late-onset hypertension. The findings indicated that women generally have a lower risk of developing cardiovascular diseases compared to men.^9^ This study sheds light on the need for tailored approaches to hypertension management based on sex. Women predominantly exhibit vagal autonomic dominance, whereas men show sympathetic nervous system dominance, particularly in HRV regulation.^10^ In addition, the variance in hypertension prevalence between sexes can be partially attributed to ovarian hormones.^11^ In fact, patients with higher BPV values are at a greater risk of hypertension-mediated organ damage^12^, implying the potential development and progression of left ventricular hypertrophy.^13^

The prevailing theory suggests that autonomic nervous system dysfunction in hypertensive patients contributes significantly to the development of cardiovascular diseases. However, the role of gender differences in this process remains underexplored and lacks systematic investigation.^1^,^9^ Mancia et al. highlighted the key updates in hypertension management, while Li et al. emphasized gender disparities in cardiovascular outcomes among elderly patients with late-onset hypertension. This study adopted a comparative approach to analyze male and female hypertensive patients, offering a gender-specific theoretical framework that advances the field of personalized medicine. By addressing these gender-specific dynamics, the cumulative findings enhance our understanding of hypertension pathophysiology and underscore the importance of tailoring diagnosis and treatment strategies to individual patients. This research not only enriches the existing theoretical models but also provides practical insights for clinical applications, advocating for personalized, gender-informed approaches to hypertension management.

Moreover, short-term BP values with increased BPV have been associated with a higher risk of cardiovascular events and all-cause mortality.^14^ While the individual correlations between BPV or HRV and primary hypertension have been extensively studied, there is limited available data on the correlation between BPV and HRV. Consequently, we investigated the correlation between BPV and HRV, as well as sex differences in primary hypertension, through simultaneous 24-hours ambulatory blood pressure monitoring (ABPM) and ambulatory electrocardiogram (Holter).

## METHODS

This retrospective cohort study examined gender differences in the relationship between BPV and HRV in patients with isolated primary hypertension, using data from Xuancheng People’s Hospital collected between December 2020 to March 2023. Patients aged 18–75 years, diagnosed with isolated primary hypertension according to the International Society of Hypertension criteria^1^, and with complete 24-hours ABPM and Holter monitoring data^9^ were included in this study. The exclusion criteria encompassed the presence of severe cardiovascular diseases (e.g., heart failure, coronary heart disease), chronic illnesses (e.g., diabetes, chronic kidney disease), use of medications affecting autonomic nervous system function, pregnancy, and incomplete data.

### Ethical approval:

It was granted by the Ethics Committee of Xuancheng People’s Hospital (Approval No.: 2024-yjky081-01, Date: April 1, 2024), and all participants provided their signed informed consent. Data analysis was conducted by an independent statistical team to ensure objectivity, with personal information anonymized to protect privacy.^3^

Comprehensive assessments, including clinical evaluations, laboratory tests, echocardiography, ABPM, and Holter monitoring, were performed. The patients were assigned to male and female groups for comparative analysis, and the results showed significant gender-specific patterns in BPV and HRV correlations. These findings offer valuable insights into the pathophysiology of hypertension and highlight the need for personalized, gender-specific management strategies.

### Data Collection:

Patient data included age, sex, history of hypertension, heart rate (HR), and baseline blood pressure. Height and weight were measured twice consecutively, and the average BMI was calculated. Left ventricular mass index (LVMI) was derived from the measurements of left ventricular end-diastolic internal diameter (LVDD), interventricular septum thickness (IVST), and posterior wall thickness (PWT) using the following formula: LVMI = {0.8×1.04× [(LVDD + LVPWT + IVST) 3 − LVDD3] + 0.6}/body surface area. Fasting blood samples were collected for total cholesterol, triglycerides (TG), serum creatinine, and plasma glucose. Synchronized 24-hours ambulatory blood pressure monitoring (ABPM) and Holter electrocardiogram monitoring were conducted using the CB-2304-A system (VasoMedical, Wuxi, China), which was calibrated prior to use. Blood pressure readings were recorded every 25 minutes during the day (7:00–22:00) and at hourly intervals at night (22:00–7:00), with valid readings exceeding 80% deemed acceptable. ABPM parameters included 24-hours systolic (24hSSD) and diastolic (24hDSD) standard deviations, coefficients of variation (24hSBPCV, 24hDBPCV), and ambulatory arterial stiffness index (AASI), which are presented in Supplementary Table-I. Holter monitoring captured 24-hours HRV data, including time-domain indices (e.g., SDNN, rMSSD, SDANN, pNN50) and frequency-domain indices (e.g., total power [TP], very-low-frequency [VLF], low-frequency [LF], high-frequency [HF]). Frequency domain data were analyzed using Fast Fourier Transform (FFT), with VLF reflecting respiratory-circulatory interactions (0.003–0.04 Hz), LF reflecting cardiac cycle modulation (0.04–0.15 Hz), and HF reflecting cardiac hemodynamics (0.15–0.4 Hz). Detailed HRV metrics are provided in Supplementary Table-II.

**Supplementary Table-I:** Parameters of Blood Pressure Variability.

Parameter	Unit	Description
24hSSD	/	Standard deviation of 24-hours systolic blood pressure
24hDSD	/	Standard deviation of 24-hours diastolic blood pressure
24hSBPCV	/	24-hours systolic blood pressure coefficient of variation
24hDBPCV	/	24-hours diastolic blood pressure coefficient of variation
AASI	/	Ambulatory arterial stiffness index

**Supplementary Table-II:** Parameters of Heart Rate Variability.

Time-domain measures				Frequency-domain measures		
Parameter	Unit	Description	Parameter		Unit	Description
SDNN	ms	Standard deviation of NN intervals	TP		ms^2^	Frequency band ≤0.4 Hz
SDANN	ms	Standard deviation of the average NN intervals for each 5-min segment of a 24-hours recording	VLFpower		ms^2^	Absolute power of the very-low-frequency band (0.0033–0.04 Hz)
rMSSD	ms	Root mean square of successive RR interval differences	LF power		ms^2^	Absolute power of the low-frequency band (0.04–0.15 Hz)
pNN50	%	Percentage of successive RR intervals that differ by >50 ms	HF power		ms^2^	Absolute power of the high-frequency band (0.15–0.4 Hz)

### Statistical Analysis:

Data were expressed as the mean ±SD for normally distributed variables or median (P25, P75) for non-normal distributions. The t-test and Kolmogorov–Smirnov test were applied for group comparisons, with Pearson’s and Spearman’s correlation tests employed for normal and non-normal data, respectively. Multiple linear regression analysis was applied to assess 24hSSD, with statistical significance set at p < 0.05. Analyses were conducted using SPSS version 22.0 (IBM, Armonk, New York, USA).

## RESULTS

A total of 189 patients, comprising 85 males and 104 females (average age: 55.06 ±12.53 years) were assessed. The male group was comparatively younger (53.85 ±13.18 vs. 56.06 ±11.94 years, *P* = 0.228) and exhibited significantly lower total cholesterol levels (4.42 ±0.76 vs. 4.71 ±0.90 mmol/L, *p* = 0.022) and higher serum creatinine levels (79.45 ±13.99 vs. 56.86 ±11.12 umol/L, *p <* 0.0*1*) and BMI (24.88 ±2.86 vs. 23.80 ±2.61 kg/m^2^, *P* = 0.007) compared to the female group. No statistically significant differences were noted in terms of age, duration of hypertension, systolic blood pressure, diastolic blood pressure, HR, triglycerides levels, plasma glucose levels, and LVMI between the two groups (*p* > 0.05) (Table-I).

**Table I:** Baseline Comparison between Male and Female Patients.

Parameter	Total	Male (n = 85)	Female (n = 104)	P value
Age, year	55.06±12.53	53.85±13.18	56.06±11.94	0.228
Duration of hypertension, month	24.0 (12.0–64.5)	24.0(9.0–70.5)	24.0(12.0–60.0)	0.515
Systolic BP, mmHg	158.22±16.16	158.11±16.24	158.31±16.17	0.932
Diastolic BP, mmHg	97.12±12.91	98.82±13.55	95.72±12.26	0.100
Heart rate, bpm	80.64±15.56	80.72±15.66	80.58±15.55	0.951
triglycerides, mmol/L	1.29(1.01–1.79)	1.34(1.03–1.81)	1.26(0.96–1.75)	0.331
Total cholesterol, mmol/L	4.58±0.85	4.42±0.76	4.71±0.90	0.022
creatinine, umol/L	67.02±16.80	79.45±13.99	56.86±11.12	<0.001
Blood glucose, mmol/L	5.24±0.53	5.20±0.56	5.27±0.51	0.387
LVMI, g/m^2^	97.86±18.40	98.27±18.17	97.52±18.67	0.780
BMI index, kg/m^2^	24.29±2.77	24.88±2.86	23.80±2.61	0.007

LVMI, Left ventricular mass index; BMI, Body mass index.

In this study, we evaluated the sex differences in BPV and HRV in 189 participants, wherein parameters such as standard deviations of systolic and diastolic pressures, AASI, and various HRV metrics like SDNN, SDANN, rMSSD, pNN50, and frequency domain measures were analyzed. Our findings indicated no significant gender-based differences for most parameters, with *p* > 0.05, suggesting similar cardiovascular variability profiles across males and females. However, a statistically significant difference was observed in HF, with *p* = 0.005, where females exhibited markedly higher HF than males (2149.37 ms² vs. 1061.79 ms²), indicating enhanced parasympathetic activity. This evidence of gender-specific cardiac vagal modulation is essential for developing tailored approaches in cardiovascular healthcare (Table-II).

**Table II:** Comparison of BPV and HRV Indicators between Male and Female Patients.

Parameter	Total	Male (n = 85)	Female (n = 104)	P value
24hSSD	14.12±3.36	14.17±3.24	14.08±3.48	0.846
24hDSD	11.03±2.67	11.36±2.69	10.76±2.63	0.124
24hSBPCV	0.10(0.09, 0.12)	0.10(0.08, 0.12)	0.11(0.09, 0.12)	0.520
24hDBPCV	0.13(0.12, 0.16)	0.14(0.12, 0.16)	0.13(0.12, 0.16)	0.459
AASI	0.38±0.18	0.38±0.18	0.37±0.18	0.723
SDNN, ms	129.26±33.28	134.31±33.98	125.13±32.28	0.059
SDANN, ms	114.21±30.88	118.18±32.15	110.96±29.57	0.110
rMSSD, ms	32.18(25.65, 44.45)	31.23(23.84, 44.26)	32.76(26.52, 44.89)	0.220
pNN50, %	4.94(2.07, 11.20)	5.51(2.17, 11.33)	4.73(2.01, 10.67)	0.461
TP, ms^2^	16720.25 (10934.65, 30133.05)	15852.04 (11031.23, 26571.74)	18194.90 (10817.17, 38398.95)	0.250
VLF, ms^2^	11944.97 (7794.66, 18459.07)	11944.97 (7612.89, 16499.63)	11869.59 (7958.53, 22895.20)	0.376
LF, ms^2^	3507.11 (1709.41, 7232.16)	3327.60 (1680.66, 6121.52)	3889.58 (1728.52, 9202.08)	0.157
HF, ms^2^	1521.86 (586.88, 3790.50)	1061.79 (492.74, 2742.25)	2149.37 (751.38, 4370.20)	0.005

24hSSD, Standard deviation of 24-hours systolic blood pressure; 24hDSD, Standard deviation of 24-hours diastolic blood pressure; 24hSBPCV, 24-hours Systolic blood pressure coefficient of variation; 24hDBPCV, 24-hours Diastolic blood pressure coefficient of variation; AASI, Ambulatory arterial stiffness index; SDNN, Standard deviation of the NN intervals; rMSSD, Root mean square of successive RR interval differences; pNN50, Percentage of successive RR intervals that differ by more than 50 ms; TP, total power; VLF, Absolute power of the very-low-frequency band (0.0033–0.04 Hz); LF, Absolute power of the low-frequency band (0.04–0.15 Hz); HF, Absolute power of the high-frequency band (0.15–0.4 Hz).

**Fig.1:**
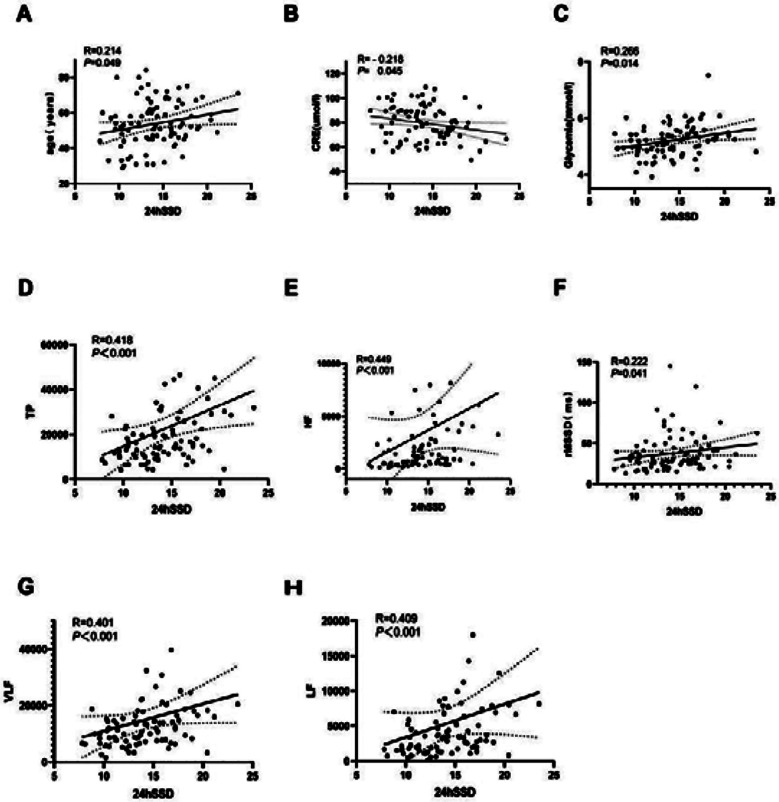
Correlation Between 24-hours Standard Deviation of Systolic Blood Pressure (24hSSD) and Various Cardiovascular Parameters in Male Patients with Primary Hypertension. Demonstration of positive correlations between 24hSSD and several parameters: A, age; B, creatinine; C, glycemia; D, total power; E, high-frequency power; F, rMSSD; G, very-low-frequency power; H, low-frequency power, highlighting 24hSSD as a marker linked to cardiovascular and metabolic variability.

**Fig.2:**
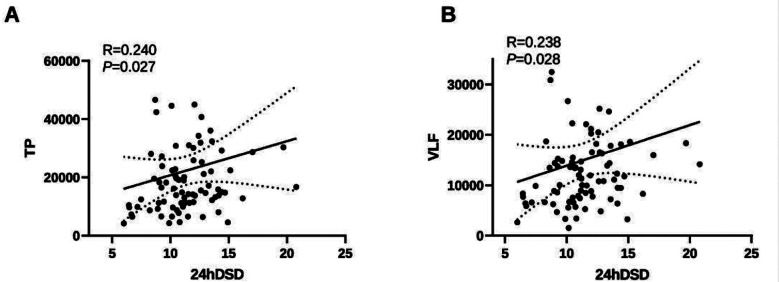
Correlation Between 24-hours Standard Deviation of Diastolic Blood Pressure (24hDSD) and Cardiovascular Parameters in Male Patients with Primary Hypertension. Demonstration of positive correlations between 24hDSD and heart rate variability parameters: A, total power; B, very-low-frequency power, indicating that higher diastolic blood pressure variability is linked to autonomic regulation.

In male patients, the univariable analysis revealed that 24hSSD was significantly positively correlated with age, plasma glucose levels, rMSSD, TP, VLF, LF, and HF (*p <* 0.05) and significantly negatively correlated with the serum creatinine levels (*p <* 0.05) (Fig.1). However, no correlation was noted between 24hSSD and the duration of hypertension, HR, triglycerides levels, total cholesterol levels, SDNN, SDANN, pNN50, LVMI, and BMI (*p* > 0.05). However, 24hDSD was significantly positively correlated with TP and VLF, but not with age, duration of hypertension, HR, triglycerides levels, total cholesterol levels, serum creatinine levels, plasma glucose levels, SDNN, SDANN, rMSSD, pNN50, LF, HF, LVMI, and BMI (*p* > 0.05) (Table-SIII, Fig.2).

**Supplementary Table-III:** Correlation Analysis of BPV in Men.

Parameter	24hSSD			24hDSD
	R-value	*P* value	R-value	*P* value
Age, year	0.214	0.049	-0.012	0.910
Duration of hypertension, month	0.043	0.693	0.063	0.565
Heart rate, bpm	0.012	0.915	-0.047	0.668
Triglycerides, mmol/L	0.055	0.619	0.002	0.985
Total cholesterol, mmol/L	0.030	0.786	-0.039	0.722
Creatinine, umol/L	-0.218	0.045	-0.088	0.423
Blood glucose, mmol/L	0.266	0.014	0.110	0.318
SDNN, ms	0.027	0.803	0.135	0.220
SDANN, ms	0.028	0.802	0.126	0.250
rMSSD, ms	0.222	0.041	0.076	0.491
pNN50, %	0.082	0.458	0.103	0.348
TP, ms^2^	0.418	0.000	0.240	0.027
VLF, ms^2^	0.401	0.000	0.238	0.028
LF, ms^2^	0.409	0.000	0.195	0.073
HF, ms^2^	0.449	0.000	0.204	0.061
LVMI, g/m^2^	0.057	0.607	0.095	0.388
BMI, kg/m^2^	0.091	0.408	0.145	0.184

24hSSD, Standard deviation of 24-hours systolic blood pressure; 24hDSD, Standard deviation of 24-hours diastolic blood pressure; SDNN, Standard deviation of NN intervals; rMSSD, Root mean square of successive RR interval differences; pNN50, Percentage of successive RR intervals that differ by more than 50 ms; TP, Total power; VLF, Absolute power of the very-low-frequency band (0.0033–0.04 Hz); LF, Absolute power of the low-frequency band (0.04–0.15 Hz); HF, Absolute power of the high-frequency band (0.15–0.4 Hz); LVMI, Left ventricular mass index; BMI, Body mass index.

**Fig. 3:**
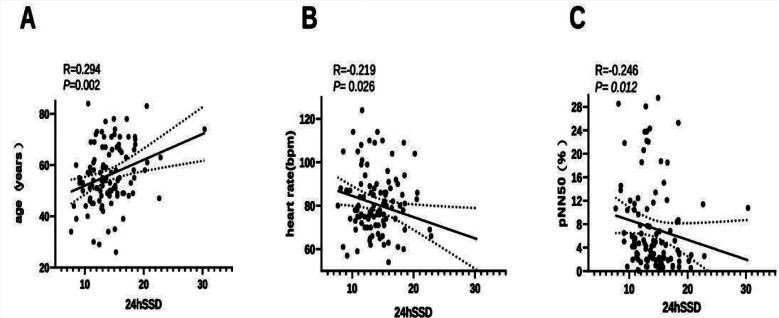
Correlation Between 24-hours Standard Deviation of Systolic Blood Pressure (24hSSD) and Cardiovascular Parameters in Female Patients with Primary Hypertension. Demonstration of correlations between 24hSSD and A, age (positive); B, heart rate (negative); C, pNN50 (negative), highlighting its link to age and autonomic function.

In women, the univariable analysis indicated that 24hSSD was positively correlated with age (*p <* 0.05) and significantly negatively correlated with HR and pNN50 (*p <* 0.05). Nonetheless, there was no correlation between 24hSSD and the duration of hypertension, triglycerides levels, total cholesterol, serum creatinine levels, plasma glucose levels, SDNN, SDANN, rMSSD, TP, VLF, LF, HF, LVMI, and BMI (*p* > 0.05) (Table-SIV, Fig.3).

**Supplementary Table-IV:** Correlation Analysis of BPV in Women.

Parameter	24hSSD			24hDSD
	R-value	P value	R-value	P value
Age, year	0.294	0.002	-0.063	0.527
Duration of hypertension, month	0.085	0.390	-0.099	0.316
Heart rate, bpm	-0.219	0.026	-0.100	0.311
Triglycerides, mmol/L	0.048	0.625	0.117	0.235
Total cholesterol, mmol/L	0.103	0.299	-0.058	0.561
Creatinine, umol/L	0.168	0.088	0.146	0.139
Blood glucose, mmol/L	0.150	0.127	-0.058	0.560
SDNN, ms	-0.127	0.200	0.067	0.498
SDANN, ms	-0.082	0.405	0.064	0.521
rMSSD, ms	-0.021	0.835	-0.020	0.839
pNN50, %	-0.246	0.012	-0.151	0.126
TP, ms^2^	-0.071	0.471	0.090	0.365
VLF, ms^2^	-0.112	0.256	0.093	0.346
LF, ms^2^	-0.074	0.453	0.057	0.564
HF, ms^2^	0.006	0.955	0.062	0.533
LVMI, g/m^2^	0.007	0.947	0.019	0.845
BMI, kg/m^2^	0.081	0.416	0.205	0.037

24hSSD, Standard deviation of 24-hours systolic blood pressure; 24hDSD, Standard deviation of 24-hours diastolic blood pressure; SDNN, Standard deviation of NN intervals; rMSSD, Root mean square of successive RR interval differences; pNN50, Percentage of successive RR intervals that differ by >50 ms; TP, Total power; VLF, Absolute power of the very-low-frequency band (0.0033–0.04 Hz); LF, Absolute power of the low-frequency band (0.04–0.15 Hz); HF, Absolute power of the high-frequency band (0.15–0.4 Hz); LVMI, Left ventricular mass index; BMI, Body mass index.

**Fig.4:**
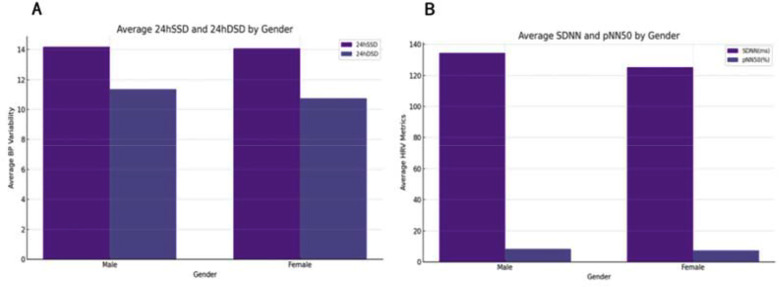
Comparison of Blood Pressure and Heart Rate Variability by Gender. Demonstration of gender differences: A, males have higher 24hSSD and 24hDSD, indicating greater BP variability; B, SDNN is similar, but females have lower pNN50, reflecting autonomic differences.

The differences in BPV and HRV between genders is illustrated in Fi4.. As shown in Fig.4A, males had slightly higher 24hSSD and 24hDSD values compared to females, indicating greater fluctuations in blood pressure among males. Fig.4B highlights HRV metrics, where SDNN exhibited minimal differences between genders, while pNN50 was slightly lower in females. These findings suggest that gender influences BPV and HRV, with notable differences in diastolic pressure variability and specific HRV parameters.

In male patients with primary hypertension, a multiple linear stepwise regression analysis was conducted with 24hSSD as the dependent variable. The age, serum creatinine levels, plasma glucose levels, rMSSD, TP (TP values are converted from logarithmic), VLF, LF, and HF were identified as independent variables, with the plasma glucose levels exhibiting the strongest correlation (Table-III).

**Table III:** Multiple Linear Regression Analysis of 24hSSD in Men.

Parameter	B	β	P Value	95%CI for B	
Constant	3.174	/	0.343	-3.453	9.802
Age, year	0.051	0.208	0.043*	0.002	0.101
Plasma glucose, mmol/L	1.436	0.247	0.017*	0.264	2.608
TP, ms^2^	3.48 × 10^-5^	0.244	0.018*	6.00 × 10^-6^	6.30 × 10^-5^

* *p* < 0.05, **p < 0.01, TP, total power.

## DISCUSSION

BPV and HRV are two vital indicators of autonomic function, whose impairment contributes to the pathomechanism of primary hypertension.^15^ BPV signifies the extent of blood pressure fluctuations within a specified period, classified into short-term and long-term variations based on the observational duration. The standard deviation of blood pressure and the coefficient of variation recorded by ABPM serve as indices for short-term blood pressure variation.^16^ Several factors influence BPV, including neuroendocrine, environmental, and behavioral factors and mechanical factors such as arterial stiffness, changes in pressure reflex sensitivity, and autonomic regulation. HRV, on the other hand, refers to temporal variations between cardiac cycles and reflects sympathetic and parasympathetic nerve functions. Increased sympathetic tone reduces HRV, whereas increased vagal tone enhances HRV.^17^

This study demonstrated significant gender-specific differences in the relationship between BPV and HRV in patients with isolated primary hypertension. Among males, the 24hSSD displayed a positive correlation with HRV parameters such as rMSSD and total power. In contrast, in females, 24hSSD was negatively associated with HR and pNN50, highlighting distinct roles of gender in autonomic nervous system regulation. These findings align with those of Mancia et al,^1^ who attributed male BPV to heightened sympathetic activation and reported that the parasympathetic neuroprotective effects were more pronounced in females.

When compared to Li et al,^9^ this study uncovered a more complex interplay between HRV and BPV in females, potentially reflecting estrogen’s regulatory influence on the autonomic nervous system. Furthermore, unlike the findings of Vasan et al,^3^ which were based on a North American cohort, this study highlights the impact of regional, racial, and demographic factors on these relationships. These results underscore the importance of tailoring hypertension management strategies to account for gender-specific physiological differences, with a focus on the interplay between BPV and HRV so as to enhance treatment efficacy and patient outcomes. In the present study, 24hSSD in BPV exhibited a significant positive correlation with rMSSD, TP, VLF, LF, and HF of HRV in the male group (*p <* 0.05). The autonomic nervous system serves as a common pathway influencing both blood pressure and HR. Sympathetic and vagal excitation and inhibition act as stabilizing factors, maintaining blood pressure and HR within the normal range. Impaired autonomic function results in disrupted sympathetic-vagal homeostasis, increased sympathetic tone, decreased vagal tone, elevated BPV, and reduced HRV.^18^

However, the present study yielded contrasting results, possibly indicating a complex correlation between the autonomic system and blood pressure changes. This autonomic reflex mechanism malfunctions in patients with isolated primary hypertension, resulting in an inverse trend, that is, higher BPV may correspond to higher HRV. In contrast, in the present study, limited correlation was noted between BPV and HRV in women, with only pNN50 demonstrating a negative correlation. Notably, a related study identified that cardiac autonomic regulation is predominantly vagal in women and sympathetic in men.

These studies often include both sexes without considering sex-specific cardiac autonomic control. Nevertheless, sex differences in primary hypertension exist, with vagal autonomic regulation prevailing in women and sympathetic autonomic regulation prevailing in men.^19^ We found that the average values of rMSSD, TP, LF, and HF indicators in the female group were higher than those in the male group in terms of HRV, while the average values of BPV indicators did not change significantly. However, they were all *p* > 0.05, indicating no statistically significant difference. This result may have been influenced by the small number of cases. However, these findings may be attributed to sex hormones, particularly estrogen, which possibly plays a pivotal role.

Furthermore, estrogen mediates autonomic regulation by enhancing vagal autonomic regulation and decreasing cardiac sympathetic autonomic regulation. Moreover, estrogen may impact vascular tone regulation and arterial blood pressure through various mechanisms, including the promotion of the release of vasodilatory, antioxidant, and anti-inflammatory factors from the endothelium.^20^,^21^ These effects collectively contribute to higher HRV in females. In addition, relevant studies have suggested that androgens influence cardiovascular autonomic control,^22^ which potentially causes vagal cardiac dysfunction in addition to ventricular dysregulation.^23^

In the present study, age was positively correlated with 24hSSD in both male and female patients with primary hypertension (R = 0.214, *p* = 0.049 vs. R = 0.294, *p* = 0.002). These findings align with those of previous studies implying that sympathetic and parasympathetic responses to primary hypertension decline with age,^24^ resulting in increased 24hSSD with advancing age. As patients age, mechanisms such as vascular sclerosis, impaired endothelial diastolic function, reduced pressure receptor sensitivity, weakening of the renin-angiotensin-aldosterone system, and diminished renal capacity to regulate water and salt may also contribute.^24^ In the present study, multiple linear stepwise regression analyses of blood pressure standard deviation in men indicated that age, glucose level, and TP were independently correlated, with the glucose level exhibiting the strongest correlation. This finding suggests that age and glucose level necessitate intensified management in the treatment of isolated primary hypertension to achieve better blood pressure control. TP is an indicator of HRV and reflects the sympathetic and parasympathetic functions. Increased sympathetic tone decreases HRV, whereas increased vagal tone enhances HRV. The autonomic nervous system is a common pathway that influences blood pressure and HR, and our study illustrates how increased TP values imply greater fluctuations in blood pressure, which indirectly reflects autonomic nervous system imbalance.

Nonetheless, our study has several limitations. First, the study did not longitudinally examine the impact of blood pressure changes on HRV, thereby failing to assess the extent to which continuous blood pressure elevation in hypertensive individuals induces pathological HRV fluctuations. Second, the study’s findings regarding sex-specific correlations between HRV and BPV slightly differ from those of previous studies. Primary hypertension is closely related to autonomic function, with the sympathetic system playing a critical role in accelerating hypertension development.^25^ In isolated primary hypertension, an increase in sympathetic involvement and/or a decrease in vagal involvement in cardiac autonomic control was notable in multiple parameters.^6^,^7^

This study analyzed the relationship between BPV and HRV in isolated primary hypertension patients based on gender differences. BPV and HRV were significantly correlated in male patients, reflecting the main regulatory role of the sympathetic nervous system, while females displayed different patterns, suggesting potential protective effects of the parasympathetic nervous system and estrogen. These findings provide a new gender-specific perspective for medical literature, particularly supported by data on Asian populations, highlighting the impact of racial and regional differences compared to that in previous studies. The research results are thus of great significance for developing personalized treatment strategies for hypertension while emphasizing the need for future research on menopausal women, racial differences, and long-term interventions in this context.

### Limitations

First, its retrospective design restricts the ability to establish causal relationships and introduces the potential for selection bias. Second, the relatively small sample size may limit the applicability of the findings to broader populations. Third, key confounding variables such as medication use, smoking habits, and other comorbid conditions that could influence BPV and HRV were not accounted for. In addition, the absence of a healthy control group hinders comparisons with baseline cardiovascular function. Finally, the study did not distinguish between premenopausal and postmenopausal women, despite the potential influence of estrogen on autonomic nervous system regulation. Future research should therefore address these gaps to provide a more comprehensive understanding of the observed associations.

## CONCLUSION

This study revealed significant differences in the relationship between BPV and HRV with respect to gender in patients with isolated primary hypertension. Male patients’ BPV is more influenced by sympathetic nervous system activation, while females exhibit a parasympathetic dominated regulatory pattern. These results emphasize the importance of considering gender specificity in hypertension management, providing a theoretical basis for developing personalized diagnosis and treatment strategies.

### Suggestions:

Further exploration of the following aspects:

1. Evaluating the regulatory role of estrogen in autonomic nervous system function;
2. Verifying the dynamic changes of BPV and HRV through prospective studies;
3. Analyzing gender differences among different ethnic and regional populations;
4. Evaluating the efficacy of gender-specific interventions in hypertension control. The knowledge of these aspects will provide more comprehensive support for precision medicine and personalized management of hypertension.

## Abbreviations:

ABPM: 24-hour ambulatory blood pressure monitoring; Holter: ambulatory electrocardiogram; BMI: body mass index; LVMI: left ventricular mass index; LVDD: left ventricular end-diastolic internal diameter; IVST: end-diastolic interventricular septum thickness; PWT: end-diastolic posterior left ventricular wall thickness; TG: triglycerides; FFT: Fast Fourier Transform.
